# New Data on the Geographical Distribution and Host Utilization of the Entomopathogenic Fungus *Myrmicinosporidium durum*

**DOI:** 10.1673/031.012.12901

**Published:** 2012-11-05

**Authors:** Sándor Csősz, Albena Lapeva-Gjonova, Bálint Markó

**Affiliations:** ^1^ Department of Zoology, Hungarian Natural History Museum, H-1088 Budapest Baross u. 13, Hungary; ^2^ Sofia University “Sv. Kliment Ohridski,” Department of Zoology and Anthropology, 8 Dragan Tzankov blv., 1164 Sofia, Bulgaria; ^3^ Hungarian Department of Biology and Ecology, Babe 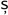 -Bolyai University, 400006 Cluj-Napoca, Clinicilor st. 5-7, Romania

**Keywords:** biodiversity, disease, fungal, host ant, parasitism, pathogen

## Abstract

Entomopathogenic *Myrmicinosporidium durum* Hölldobler, 1933, a fungus known to exploit several ant species, is reported for the first time in five countries: Bulgaria, the Czech Republic, Romania, Slovakia, and Turkey. The discovery of the fungus in Anatolia significantly widens its known distribution. In addition, this fungal parasite was found to utilize two hitherto unknown host species: *Tetramorium* sp. D (*sensu* Schlick-Steiner et al. 2006) and *Tetramorium* sp. E (*sensu* Schlick-Steiner et al. 2006). According to the new data, *M. durum* seems to be more common in Europe than previously thought, while its host range is considerably larger. In the present paper, data on its currently known distribution and host preference are discussed.

## Introduction

The enigmatic obligate entomopahtogenic fungus *Myrmicinosporidium* durum Hölldobler, 1933 is known to infect various ant species across Europe and America (Sanchez-Peña et al. 1993; Buschinger et al. 2004; Pereira 2004; Espadaler and Santamaria 2012). This fungal parasite is responsible for infections that are characterized by the presence of darker, lentiform capsules (∼ 30–60 µm in diameter), and the thick-walled fungal spores can be spotted through the cuticle under a microscope (Buschinger et al. 2004; Gonçalves et al. 2012). Though *M. durum* was discovered about 80 years ago, and plenty of data are available on its distribution and host preference, information on its effect on the host is scarce (see Espadaler and Santamaria 2012), and its systematic position has still not been decisively cleared up.

Salient features of the general biology of *M. durum* were outlined by Hölldobler (1927, 1933), and later some details of species description were reviewed (Espadaler 1982; Buschinger and Winter 1983; Sanchez-Peña et al. 1993). Though a considerable amount of information about its host utilization and geographic distribution is available (Pereira 2004; Espadaler and Santamaria 2012), these characteristics are poorly understood. Upon enlisting its host species, *M. durum* appears to be a generalist parasite, known to infect 35 ant species in three different subfamilies (Gonçalves et al. 2012).

Contrary to what the long list of host species might seem to suggest, the life-cycle and dispersal strategies of *M. durum* have remained almost completely unknown. The mature spores of the fungi can be detected quite commonly in the gaster of lightly colored host ants, most frequently in the fall and early spring (Sanchez-Peña et al. 1993; Buschinger et al. 2004).

Though for a long time, this parasitic fungus has received scant consideration in myrmecological discussions, in the recent years it has been drawing the attention of specialists (Buschinger et al. 2004; Garcia and Espadaler 2010; Espadaler and Santamaria 2012; Gonçalves et al. 2012). The increasing scientific interest requires extensive knowledge of host utilization and well-supported data on the biogeography of the fungus. This information may bring a better understanding of host-parasite interactions and may additionally clarify the background of other fungal parasites phenotypically similar to *M. durum.*

The latest comprehensive reviews (Garcia and Espadaler 2010; Espadaler and Santamaria 2012) give summarized data of localities, from the Galápagos (Ecuador), the U.S.A., and eight southern, western, and central European countries; eastern Europe remained a blank area in this regard. The geographical bias in the distribution data of the fungus (e.g., lack of information regarding Eastern Europe and Asia) calls for additional studies. In this study, new data are presented on the presence of *M. durum* in Eastern and Southern Europe and for the first time in Asia (Asian part of Turkey).

In addition to the new distribution data, new information regarding two new host species is provided, which further widens the host preference of this fungal parasite, laying emphasis on the non-selective character of its host choice.

## Materials and Methods

Extensive faunistic surveys were conducted by the authors in several Central, East, and South European and Turkish sites over the course of a four-year (2008–2012) period. Samples were collected by hand-searching and by means of pitfall traps. Collected material was stored in separate vials with 96% EtOH and transported to the laboratory, where the infestation was later observed. *Tetramorium* ants were determined by the first author.

## Results and discussion

**Figure 1.**
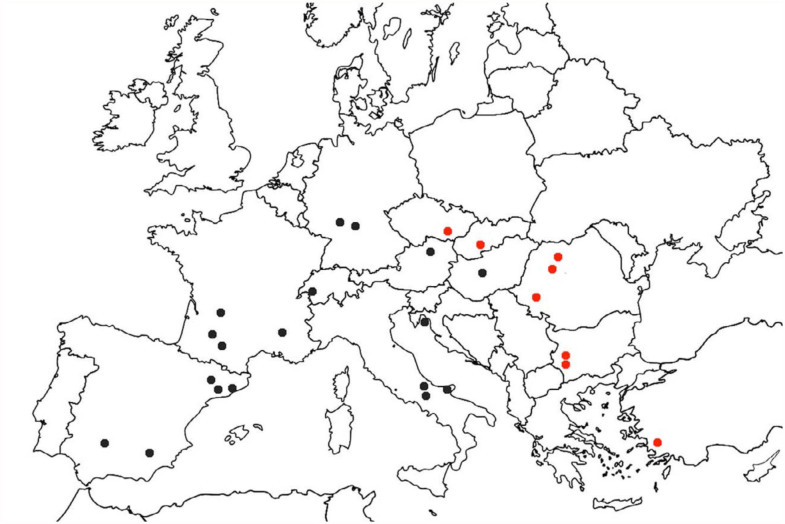
Distribution of collecting sites of new *M. durum* occurence data (red points) and previously known localities (black points). High quality figures are available online.

Altogether 21 infected worker specimens belonging to six different ant species were found in eight samples from four European countries and the Asian part of Turkey (Table 1, Figure 1). Two ant species, *Tetramorium* sp. D and *Tetramorium* sp. E (*sensu* Schlick-Steiner et al. 2006), are reported for the first time as hosts of *M. durum.*

**Figure 2.**
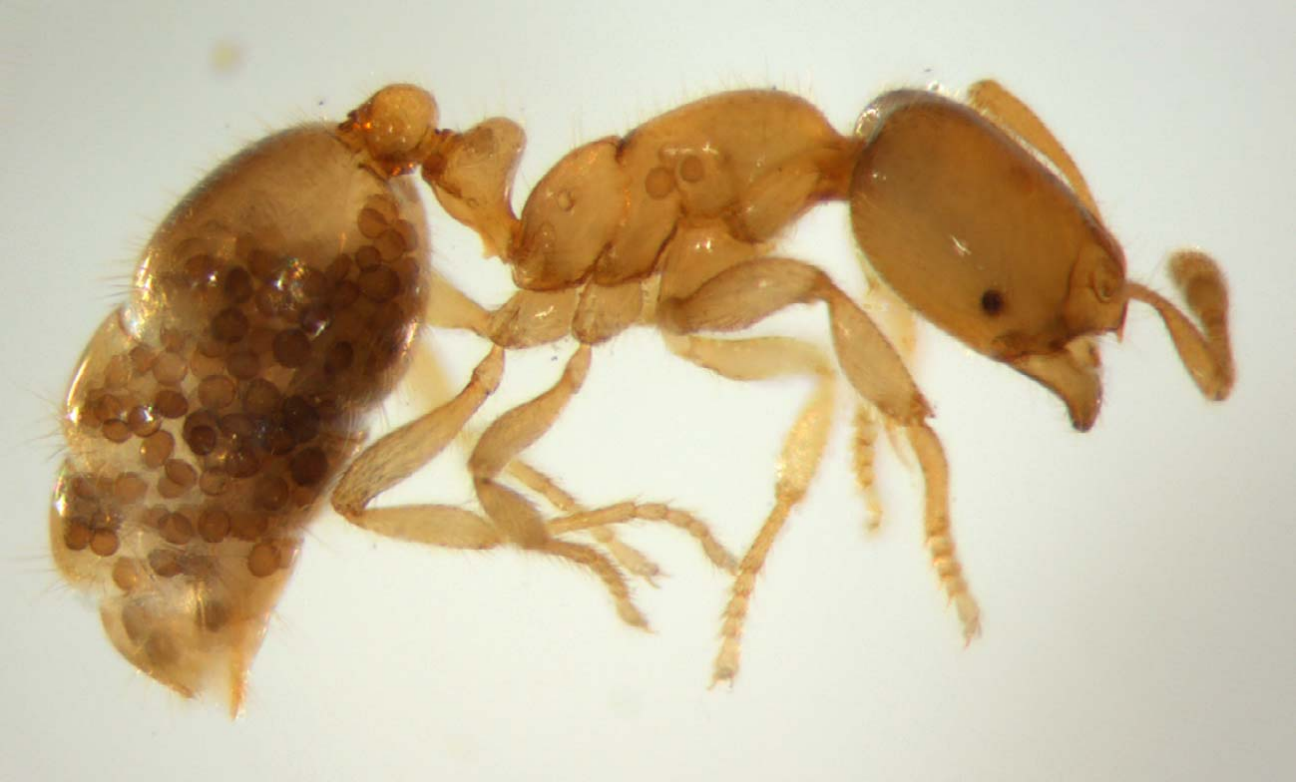
Mass of *M. durum* spores are visible in the gaster and the mesosoma of *Solenopsis fugax* worker collected in Czech Republic, near Mikulov at the end of August, 2010. High quality figures are available online.

These new findings are not surprising when the hard detectability of infestation is taken into account. Sixteen of all affected ants belonged to the *Solenopsis* genus, which is one of the most frequently reported hosts for this fungal parasite (see Espadaler and Santamaria 2012) because the spores are more visible through the lightly colored integument (Figure 2). In this study, most of the infected ants were collected in a tight window from August to October, and only two samples were collected in March. According to the new records, *M. durum* is known from America and is well-represented across Europe; its distribution stretches from Portugal (Gonçalves et al. 2012) to the easternmost shores of the Mediterranean Basin.

**Table 1.**
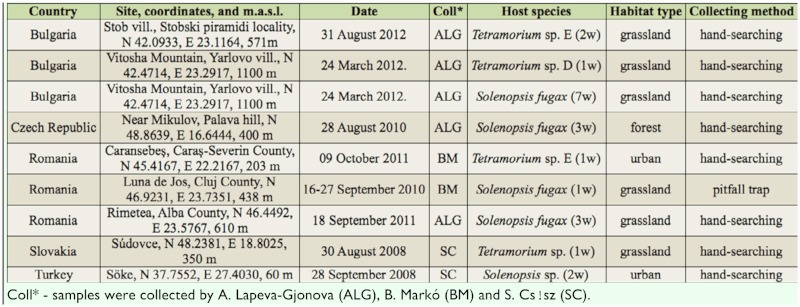
List of locations where *M.* *durum* infestation was discovered.

The puzzling overall picture of the wide distribution of this fungal species prompted the provision of an alternative explanation. The question is posed as to whether the observed wide geographic distribution can be ascribed to a cryptic diversity. This broadly defined fungal species can still be recognized solely on the basis of gross morphological characteristics, which raises the possibility that more phylogenetic lineages of fungal parasites phenotypically similar to *M. durum* might have been lumped within this phenetically circumscribed entity. Questions concerning the fungus' systematic position should be answered with the help of molecular genetic or diagnostic methods, but in order to obtain a better understanding of the biology of this enigmatic organism, information on its distribution, life cycle, or pathogenic nature is essential.

Hence, the new data provided in this study on hitherto unexplored localities and hosts helps in accumulating knowledge about this life form, which may later form the basis of further questions on the complex life cycle of this enigmatic fungal species.
